# Contribution of greenhouse gas footprint of broiler product during electrical slaughtering in Taiwan

**DOI:** 10.1016/j.psj.2025.105528

**Published:** 2025-07-05

**Authors:** Chi-Yu Chuang, Jui-Hong Hsiao, Min-Chuan Lai, Jui-Lin Hsu, Chung-Hsi Chou

**Affiliations:** a Center of Corporate ESG and Sustainability Research, Chang Jung Christian University, Tainan City 711, Taiwan; b Animal and Plant Health Inspection Agency, Ministry of Agriculture, Taipei City 100, Taiwan; c Zoonoses Research Center and School of Veterinary Medicine, National Taiwan University, Taipei City 106, Taiwan

**Keywords:** Greenhouse-gas emissions, Electrical slaughterhouses, Broiler

## Abstract

The food system is responsible for nearly one-third of global greenhouse gas (GHG) emissions, with broiler production having a significant impact. Electrical slaughtering is the common method for producing broiler products in industrialized countries, but there has been insufficient research to measure its GHG footprint. Following the ISO 14067 standard for carbon footprint quantification of product, this study collected activity data (electricity utilization, refrigerant filling, fossil fuel consumption and wastewater management) during 2021 from two representative poultry electrical slaughterhouses in Taiwan to determine the GHG footprint contribution of broiler products during electrical slaughtering. The results show that electricity consumption contributes the most, accounting for 62.5 % to 71.7 % of the GHG footprint in the slaughterhouses. Refrigerant use (approximately 6.0 % to 11.7 %) and fossil fuel utilization (approximately 6.9 % to 8.5 %) are minor emission sources. Methane generated from wastewater treatment contributes about 5.0 % to 21.6 % of the GHG footprint, varying with pollutant load, and it is recommended that slaughterhouses carefully control this aspect.

## Introduciton

Meat consumption plays a crucial role in the contemporary human diet. However, as the global population keeps escalating, it can be anticipated that the consumption of meat will persistently augment, exacerbating the environmental ramifications. In 2015, the global greenhouse gas emission (GHG) emissions from the food system attained 18 billion tons of carbon dioxide equivalent (CO₂e) per annum, constituting 34 % of GHG emissions(Crippa et al., 2021). The largest sources of GHG emissions originated from agricultural production, land use and its change (Crippa, et al., 2021). The related impacts of animal farming are even more prominent. The same product can be produced by different producers through diverse means, and the disparity in the calculation results of the generated GHG emissions can sometimes reach up to 50 times. How to optimize the meat production methods to reduce GHG emissions and alleviate the environmental impact has become a vital topic (Mostert, et al., 2022).

In order to decrease the GHG emissions in meat production, it is necessary to understand the GHG emission hotspots, through the product carbon footprint and life cycle assessment, to understand the GHG emissions in different stages of the entire production process. Based on the existing meat life cycle assessment studies, it can be found that the GHG emissions in the manufacturing stage (feeding, slaughtering and processing) are the most impactful (Akamati, et al., 2023; Prudêncio da Silva Jr, 2011).

However, prior studies on GHG emissions have largely overlooked the emissions associated with electrical slaughtering systems in poultry processing—an industrialized and widely adopted practice in developed countries known for its efficiency and scalability. This omission presents a gap in the current literature, particularly in evaluating the environmental impacts of modern slaughtering technologies. A comprehensive analysis of the emissions arising from the electrical slaughtering process is therefore essential for understanding Taiwan’s poultry sector emissions profile and for developing targeted mitigation strategies. In this study, we collected actual field data GHG from electrical slaughterhouses. This data was used to calculate the annual GHG emissions, enabling us to estimate the GHG footprint contribution of the final broiler product.

## Material & methods

### Target broiler electrical slaughterhouses selection

This study included the two broiler chicken electrical slaughtering houses (named as slaughterhouse SH A and SH B) as targets in Taoyuan City, north Taiwan. These two targets are sized similarly, as well as same broiler processing stages before the final products. Both broiler slaughterhouse SH A (annual capacity over 18 million pieces, average weight 2.20 kg/pieces) and SH B (annual capacity over 20 million pieces, average weight 2.09 kg/pieces) are listed as top 10 electrical slaughtering plants among all 118 plants in Taiwan. The final slaughtered products are white broiler chickens, which have undergone electrical slaughtering procedures such as electrical stunned, air-cooling bloodletting, scalding, feather removal, evisceration, final treatment, cooling, cutting and packaging, refrigeration and freezing, and including the transportation and disposal of waste generated in this process.

### GHG emissions calculation of target broiler electrical slaughterhouses

The calculation of GHG emissions in this study was followed with ISO standard 14067:2018 (Verification –Greenhouse Gases –Carbon Footprint of Products) and Operation Direction for GHG Validation and Verification Bodies Management (announced by Ministry of Environment (2023)). The system boundary was set as gate-to-gate of individual target slaughterhouses and compliant with the carbon footprint product category rules published by the Ministry of Environment, Taiwan (No.19-014, livestock, poultry and edible offal products, version 2.0, 2019).

**Fig. 1:**
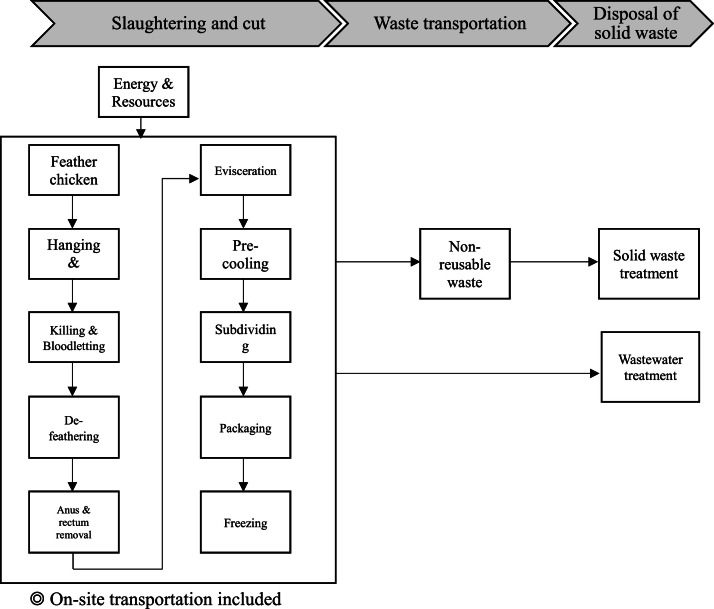
The schematic diagram of system boundary of electrical slaughtering in target slaughterhouse.

Following with the direction and protocol described above, the system boundary encompassing the major stages of processing, including (A) slaughtering and cut, (B) waste transportation and (C) disposal of solid waste. The solid waste and wastewater generated during the process of slaughtering, cutting, and packaging of frozen broiler products was also included in the system boundary, as shown in Fig. 1.

Four relevant kinds of GHG were identified and calculated in target electrical slaughterhouses of this study, including carbon dioxide (CO_2_), nitrous oxide (N_2_O), methane (CH_4_), hydrofluorocarbons (HFCs) and converted to carbon dioxide equivalent (CO_2_e) among total seven GHG (listed as before, plus perfluorocarbons (PFCs), sulfur hexafluoride (SF_6_) and nitrogen trifluoride (NF_3_)) in operation direction. This study collected activity data based on actual annual consumption of electrical power, refrigerant and fossil fuels. All the fossil fuel-driven utility bills (including liquefied natural gas, liquefied petroleum gas, and diesel oil), the nomenclature plates of electrical utilities (recording the theoretical power output), as well as the estimation of refrigerant used and leaks were included. The aforementioned data covered the study period from January 1, 2021, to December 31, 2021. The GHG emissions activity data for both target slaughtering houses are shown in Table 1.

**Table 1:** GHG emission source identification and activity data collection of target slaughterhouse SH A and SH B.

	SH A			SH B		
Stage	GHG Source	Machine or Equipment	Notes	GHG Source	Machine or Equipment	Notes
Slaughtering / Cutting	LNG	Boiler	The fuel switched from diesel to LNG in May, 2021	LPG	Boiler	Calculated according to the sub-meter of LPG in slaughterhouse
	Diesel	Boiler		Diesel	On-site machinery and equipment, transportation tools and generators	There is no separate records of diesel distribution to generators and equipment; assuming that all are used by forklifts.
	Diesel	On-site machinery and equipment, transportation tools and generators	This year the generator is not in operation, all are used by the forklifts.			
	Gasoline	Company car	-	Gasoline	Company car	-
	Refrigerant	Refrigeration equipment	Total **42** large equipment	Refrigerant	Refrigeration equipment	Total **22** large equipment
	Power	On-site power consumption	Electrical slaughter house and food processing plant	Power	On-site power consumption	There are 3 sub-meter for power calculation.
	Water	On-site water consumption	Electric slaughter house and food processing plant	Water	On-site water consumption	
Waste transportation	Waste transportation	Organic sludge	-	Waste transportation	Solid waste	-
		General waste	-			
		Chemical mixture	Very small amount about 12kg			
Waste disposal	Waste disposal	Above mentioned waste	Incineration	Waste disposal	Solid waste	Incineration
		Wastewater treatment	Aerobic treatment	Waste disposal	Wastewater treatment	Aerobic treatment

The calculation of the GHG footprint is using the assumption of activity data multiplied with emission factors and global warming potential (GWP, only for refrigerant filling of refrigeration and cooling equipment) follow with the equation:

$$\text{GHG}\;\text{footprint}=\sum_{i=1}^{n}\left(AD_{i}\times EF_{i}\times GWP\right)$$

Where GHG footprint is GHG emission of final broiler product (in kg CO₂e, GWP = 1). *AD_i_* is activity data (e.g., electricity utilization, refrigerant filling, fossil fuel consumption, waste disposal and wastwewater drainage). *EF_i_* means GHG emission factor corresponding to the various activities (in kg CO₂e per unit of activity, shown in Table 2). In addition, *n* represents total number of individual activities included within the system boundary. The GWP values (between 0.003 ∼ 0.16, varied from various equipment) utilized in this study is following with the latest official 2019 version of Ministry of Environment, Taiwan, shown in Table 3).

**Table 2:** Source and emission factor of GHG in target slaughterhouses applied.

Emission Factor	value	unit	Reference
Gasoline for passenger car, 2020	3.010	kgCO_2_e/L	Carbon Footprint Information Platform, Ministry of Environment, Taiwan
Diesel for stationary sources.,2020	3.340	kgCO_2_e/L	
Diesel for rail and non-road transportation and,2020	3.610	kgCO_2_e/L	
Natural Gas for stationary sources,2020	2.630	kgCO_2_e/m^3^	
LPG for stationary source, 2020	2.220	kgCO_2_e/L	
GHG footprint of electricity generation, 2020	0.590	kgCO_2_e/kwh	
GHG footprint of tap water generation, 2017	0.299	kgCO_2_e/m^3^	
Diesel for heavy truck (over 3.5 ton)	0.235	kgCO_2_e/tkm	
Diesel for light truck (less than 3.5 ton)	0.647	kgCO_2_e/tkm	
Diesel for waste truck transportation	1.310	kgCO_2_e/tkm	
waste incineration	360.0	kgCO_2_e/ton	
R134A refrigerant production	16.20	kgCO_2_e/kg	SimaPro version 9.0.4, PRé Sustainability
CH_4_ generation of wastewater treatment of industry	0.250	Kg CH_4_/kg COD	National Greenhouse Gas Inventory, Ministry of Environment, Taiwan, 2022
Methane correction factor (aerobic)	0.05	−	
Methane correction factor (anaerobic)	0.20	−

**Table 3:** Emission factor of refrigeration equipment.

Item	Source	Emission Factor (kg/kg)	Emission Factor Source
Refrigeration and Cooling Equipment	Household Cooling equipment	0.003	GHG Emission Factor Management Table Version 6.0.4. Ministry of Environment, Taiwan, 2019
	Industrial freezing and refrigeration equipment, including food processing and refrigeration	0.160	
	Water chiller	0.090	
	Air conditioners for residential and commercial buildings	0.030	

## Results

### GHG footprint of broiler product from target slaughterhouse SH A and SH B

**Fig. 2:**
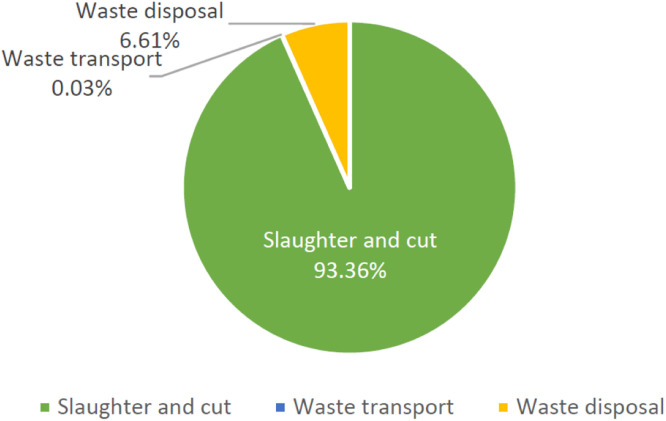
The proportion of carbon footprint at each stage of the slaughtering process in SH A.

In this study, the actual activity data and emission factors of four kinds of GHG from two target slaughterhouses were collected to calculate annual GHG emission for estimating the contribution of GHG footprint of final broiler product, the results of total GHG emission of target slaughterhouses is shown in Tables 4 and 5. According to the results of the system boundary carbon footprint in this study, the total emission of slaughterhouse SH A is 7,231.190 ton CO₂e. Based on the annual slaughtered amount 18,246,909 pieces in 2021, the unit GHG footprint of slaughtering broiler product is 0.180 kg CO₂e /kg. The GHG footprint of per chicken slaughtered is 0.396 kg CO₂e/piece, as shown in Table 6. Slaughtering and cutting stage accounted for 93.36 % of the total GHG footprint of each stage, followed by wastewater treatment stage accounted for 6.61 %, and waste transportation stage accounted for only 0.03 %. In the slaughtering and cutting stage, the main source of GHG emission is came from electricity power, accounts for 76.86 %. Refrigeration equipment refrigerant and LNG accounting for 12.59 %, and 7.47 %, respectively. The above carbon emission accounts for 96.62 % in the slaughtering and cutting stage, as shown in Fig. 2.

**Table 4:** GHG emission for each stage of the electrical slaughtering process in SH A.

Stage	Equipment	Source	value	unit	GHG emission (tCO_2_e)	Proportion(%)
Slaughtering / Cutting	Boiler	LNG	191,733	m^3^	504.258	6.97 %
	Boiler	Diesel	45,000	L	150.30	2.08 %
	On-site machinery and equipment, transportation tools and generators	Diesel	4,309	L	15.555	0.22 %
	Company Car	Gasoline	9,857	L	29.670	0.41 %
	Refrigeration equipment	Refrigerant R22	395.446	kg	781.481	10.81 %
		Refrigerant R404A	14.304	kg	67.861	0.94 %
		Refrigerant R410A	0.276	kg	0.627	0.01 %
		Refrigerant R32	0.138	kg	0.109	<0.01 %
	Power	Power	8,794,865	kWh	5,188.97	71.76 %
	Water	Water	41,792	m^3^	12.496	0.17 %
Waste transportation	Waste transportation	Organic sludge	5,974.51	tkm	1.404	0.02 %
		General waste	619.05	tkm	0.811	0.01 %
		Chemical mixture	4.008	tkm	0.003	<0.01 %
Waste disposal	Waste disposal	Organic sludge	196.53	ton	70.751	0.98 %
		General waste	108.60	ton	39.098	0.54 %
		Chemical mixture	0.012	ton	0.004	<0.01 %
		Wastewater treatment (with power)	322,935	kWh	190.532	2.63 %
		Wastewater treatment (methane)	508,272	kgCOD	177.260	2.45 %
Total					7,231.190	100%

**Table 5:** GHG emission for each stage of the electrical slaughtering process in SH B.

Stage	Equipment	Source	value	unit	GHG emission (tCO_2_e)	Proportion(%)
Slaughtering / Cutting	Boiler	LPG	111,575	kg	450.312	8.59 %
	On-site machinery and equipment, transportation tools and generators	Diesel	3,000	L	10.83	0.21 %
	Company Car	Gasoline	3,540	L	10.658	0.20 %
	Power	Power	5,555,372	kWh	3,277.66	62.53 %
	Water	Water	29,80	m^3^	8.912	0.17 %
	Refrigeration equipment	Refrigerant R22	124.36	kg	245.774	4.69 %
		Refrigerant R410A	1.087	kg	2.469	0.05 %
		Refrigerant R507A	12.00	kg	57.494	1.10 %
		Refrigerant R32	0.097	kg	0.077	<0.01 %
		Refrigerant R134A	0.002	kg	0.003	<0.01 %
		Refrigerant R404A	1.896	kg	8.995	0.17 %
Waste transportation	Waste transportation	General solid waste	5,550.48	tkm	7.271	0.14 %
Waste disposal	Waste disposal	Wastewater treatment (with power)	380,179	kWh	224.306	4.28 %
		Wastewater treatment (methane)	652,006.985	kgCOD	909.55	17.35 %
		General waste	77.090	ton	27.752	0.53 %
Total					5,242.072	100%

**Table 6:** GHG emissions per unit of broiler product in SH A.

Item	value	unit	Unit GHG emission (kgCO_2_e/unit)
Feather chicken weight	40,136,095	kg	0.180
Number of feather chicken	18,246,909	-	0.396

**Fig. 3:**
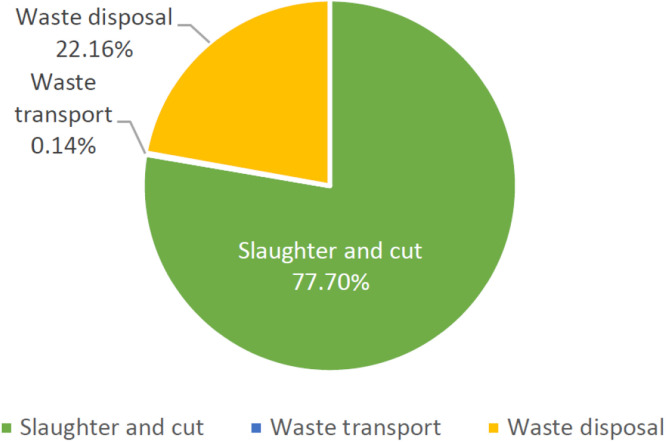
The proportion of carbon footprint at each stage of the slaughtering process in SH B.

On the other hand, in the case of slaughterhouse SH B, the total GHG emission was 5,242.072 ton CO₂e. The unit GHG emission of slaughtering broiler product is 0.120 kg CO₂e /kg. The GHG footprint of per chicken slaughtered is 0.252 kg CO₂e/piece based on 2021 annual 20,835,775 pieces slaughtered, as shown in Table 7. Slaughtering and cutting stage accounted for 77.70 % of the GHG footprint of each stage, followed by the wastewater treatment stage accounted for 22.16 %, and the waste transportation stage accounted for only 0.14 %. The analyzation of GHG footprint from each project in slaughtering and cutting stage indicate that the main source of electricity accounts for 80.47 %, followed by LPG accounting for 11.06 %, refrigerant accounting for 7.73 %, and the above GHG emission accounts for 99.25 % of the total slaughtering and cutting stage, as shown in Fig. 3.

**Table 7:** GHG emissions per unit of broiler product in SH B.

Item	value	unit	Unit GHG emission (kgCO_2_e/unit)
Feather chicken weight	43,546,707	Kg	0.120
Number of feather chicken	20,835,775	-	0.252

In this study, slaughterhouse SH A presents about 1.38 times higher total GHG footprint than SH B (1,989 ton CO_2_e). On the other hand, slaughterhouse SH A was found to produce about 1.50 and 1.57 times higher GHG footprint based on broiler product per kg weight unit (SH A = 0.180 and SH B = 0.120 kg CO₂e /kg). Slaughterhouse SH A also have 1.57 times higher GHG footprint while counting based on per chicken slaughtered (SH A = 0.396 and SH B = 0.252 kg CO₂e /piece).

## Discussion

In 2021, Taiwan's total chicken meat production was estimated at approximately 688,000 metric tons, reflecting a consistent supply to meet both domestic consumption and industrial demand. Per capita poultry meat consumption in the same year reached 39.94 kilograms, positioning Taiwan among the higher-consuming nations globally in terms of chicken meat intake. The estimated total GHG emissions of chicken industry were approximately 3.8 to 4.4 million tons of CO₂e. In 2021, Taiwan's chicken meat self-sufficiency rate reached 88 %. In Taiwan, where over 90 % of poultry slaughtering is conducted using electrical stunning in centralized slaughterhouses, the lack of detailed emissions data from this stage presents a significant obstacle to accurate life cycle assessment (LCA) and carbon footprint estimation. (Council of Agriculture, 2022), indicating that the majority of the carbon emissions associated with chicken meat production are generated domestically, particularly during the stages of rearing, slaughtering, and processing. Although feed remains a major source of upstream emissions and is largely imported—Taiwan depends on imports for over 95 % of its soybean and maize feed ingredients (Ministry of Agriculture, Taiwan, 2022) — the in-country production chain still contributes significantly to the overall carbon footprint. In particular, emissions from the slaughtering phase, especially electrical stunning, cutting, refrigeration, wastewater treatment, and packaging, are critical components of the post-farm gate emission profile. Analyzing the carbon sources in the slaughtering process is therefore essential for identifying domestic emission hotspots and formulating effective mitigation strategies tailored to Taiwan and also Asia poultry sectors.

Accord to annual GHG emission estimation of final broiler product during electric slaughtering in this study. For target slaughterhouse SH A, the largest source (hotsopt) of emissions is electricity usage, followed by refrigerant filling, then fossil fuel usage, and finally waste treatment (including transport and disposal), as shown in Table 8. Instead, the hotspot of emissions is electricity usage but followed by wastewater treatment, then fossil fuel usage and refrigerant filling in slaughterhouse SH B, as shown in Table 9.

**Table 8:** Target Slaughterhouse SH A’s emission sources and hot spot.

No.	Source	value	unit	GHG emission (tCO_2_e)	Proportion(%)	
1	Power	8,794,865	kWh	5,188.97	71.76 %	71.76 %
2	Refrigerant R22	395.446	kg	781.481	10.81 %	11.76 %
	Refrigerant R404A	14.304	kg	67.861	0.94 %	
	Refrigerant R410A	0.276	kg	0.627	0.01 %	
	Refrigerant R32	0.138	kg	0.109	<0.01 %	
3	LNG	191,733.237	m^3^	504.258	6.97 %	6.97 %
4	Wastewater treatment (With power)	322,935	kWh	190.532	2.64 %	5.09 %
	Wastewater treatment (methane)	508,272	kgCOD	177.260	2.45 %	
Total				6,911.098	95.57 %	95.57 %

**Table 9:** Target slaughterhouse SH B’s emission sources and hot spot.

No.	Source	value	unit	GHG emission (tCO_2_e)	Proportion(%)	
1	Power	5,555,372	kWh	3,277.669	62.53 %	62.53 %
2	Wastewater treatment (With power)	380,179	kWh	224.306	4.28 %	21.63 %
	Wastewater treatment (methane)	652,006.985	kgCOD	909.55	17.35 %	
3	LPG	111,575	kg	450.312	8.59 %	8.59 %
4	Refrigerant R22	124.367	kg	245.774	4.69 %	6.01 %
	Refrigerant R410A	1.087	kg	2.469	0.05 %	
	Refrigerant R507A	12.0	kg	57.494	1.10 %	
	Refrigerant R32	0.097	kg	0.077	<0.01 %	
	Refrigerant R134A	0.002	kg	0.003	<0.01 %	
	Refrigerant R404A	1.896	kg	8.995	0.17 %	
Total				5,176.649	98.75 %	98.75 %

Target slaughterhouse SH A was found to have a larger GHG footprint both on per chicken slaughtered and final broiler weight than SH B. Equipment design and application result in differences in GHG emissions from power utilization, refrigerant leakage, fossil fuel consumption and waste disposal between target slaughterhouses.

### GHG emissions from electricity utilization and refrigerant filling between target slaughterhouse SH A and SH B

Although slaughterhouse SH B has a higher annual processing capacity than SH A (20 million vs. 18 million birds), it demonstrates superior carbon efficiency. The greenhouse gas (GHG) emissions per chicken slaughtered are significantly lower at SH B, with an emission factor of 0.252 kg CO₂e per bird compared to 0.396 kg CO₂e at SH A. This trend is also reflected in the carbon footprint per unit of final broiler product, where SH B reports 0.120 kg CO₂e per kg of meat, substantially lower than SH A’s 0.180 kg CO₂e/kg. In addition, the GHG footprint of unit final broiler product between SH A and SH B reveal the same trend (0.180 and 0.120 kg CO₂e/kg, respectively). Comparing the GHG emission sources between slaughterhouse SH A and SH B, the electricity was found to attribute the difference. Electricity consumption of slaughterhouse SH A is higher than that of slaughterhouse SH B (8,794,865 and 5,555,372 kWh, respectively).

Analyzing the differences between these two slaughterhouses in power electricity consumption, the number of refrigeration equipment and cooling process setting were found to be major factors. Slaughterhouse SH A operates 42 refrigeration equipment, nearly double the number found in SH B, which utilizes 22 equipment This disparity contributes to substantially higher electricity consumption associated with product refrigeration at SH A. Moreover, the total refrigerant charge—identified as the second-largest source of greenhouse gas emissions in both facilities—is notably higher in SH A. Both slaughterhouses predominantly use R-22 as the primary refrigerant (following with the operation manuals of each equipment, shown in Table 4, Table 5). The environmental impact is further exacerbated by refrigerant leakage rates, with SH A reporting a leakage rate of 11.76 %, almost twice that of SH B’s 6.01 %. These findings underscore the critical role of refrigeration system design and maintenance in managing both direct and indirect emissions in poultry processing operations.

Focusing on the electricity usage of cooling process, SH A utilize air-cooling tunnel (length: 85 m, piece moving for 6 minute, temperature range: −2 ∼ 2 °C) for chilling the heat before cutting, which is not equipped in slaughterhouse SH B. In addition, the pre-cooling procedure may also be the reason that caused higher electricity consumption. slaughterhouse SH A is equipped with four tanks, which is more three tanks in SH B. Besides, the operation setting of pre-cooling tanks (length, residence time and temperature) in slaughterhouse SH A may also cause more power usage than SH B (shown in Table 10).

**Table 10:** The pre-cooling tanks and setting of target slaughterhouse SH A and SH B.

Slaughterhouse	Pre-cooling tank	Length	Time	Temperature
SH A	I	5 m	10 min	20 °C
	II	10 m	35 min	4 °C
	III	6.3 m	15 min	4 °C
	IV	6 m	15 min	3 °C
SH B	I	3 m	5 min	23 °C
	II	7.3 m	20 min	17 °C
	III	11 m	30 min	2 °C

The GHG emission comes from electricity consumption depends on the composition of electricity generation. Raising the proportion of natural gas and renewable sources (such as solar panel and wind turbine), rather than coal and oil may reduce the GHG emission of electricity per unit. In this study, 0.590 kg CO_2_e/kwh was set to calculate the GHG emission in slaughterhouse according to 2020 electrical carbon footprint coefficient, announced by Ministry of Environment, Taiwan. Previous studies indicate that electricity carbon emission of China is about 1.03 kg CO_2_e/kwh (caused by high proportion of coal fire power), result in higher global warming impaction during slathering stage (0.11 kg CO_2_e/kg, in poultry live weight) of boiler product (Cheng, et al., 2023). As for France, which use near 70 % nuclear power, has the electricity carbon emission low to 0.146 kg CO_2_e/kwh and result in much less global warming impaction (0.05 kg CO_2_e/kg, in poultry live weight) (Prudêncio da Silva Jr, 2011). In this study, the global warming impaction of target slaughterhouse SH A and SH B are 0.18 and 0.12, respectively. Decrease the electrical carbon footprint is the vital to slaughterhouses for produce greener products. Distributed power generation system, including solar photovoltaics, wind turbine, biomass co-generation is encouraged to install in the agricultural facility for gaining more green energy. Energy storage system is also helpful in providing more flexibility and safety of power utilization.

In the study of Vitali et al., 2018, the management of refrigeration equipment was essential to save energy. To ensure energy efficiency during the shipment of meat product, it is crucial to prevent the temperature of refrigeration from rising excessively, as this would necessitate re-cooling and consequently increase electricity consumption. Regular inspections of the refrigeration equipment 's sealing performance, door condition, and operational status of the refrigeration equipment should be conducted to ensure proper door sealing and the absence of air leaks. Additionally, reducing the frequency and duration of freezer door openings will help minimize the loss of cold air. Furthermore, accurately calculating the storage requirements of the refrigeration and freezing units to avoid overfilling is essential to ensure even air circulation and reduce the risk of temperature increases within the units. Installing an effective monitoring system to continuously track the temperature of the refrigeration and freezing units is also recommended. If the temperature exceeds the preset range, an alarm should be triggered to prompt immediate action (Vitali, et al., 2018).

### GHG emissions from fossil fuel consumption between target slaughterhouse SH A and SH B

The fossil fuel is the second and third largest sources of GHG emission after power electricity in target slaughterhouse SH A. In the slaughterhouse SH A, 9.26 % of GHG emission comes from fossil fuel consumption. 8.59 % of GHG emission comes from fossil fuel consumption in slaughterhouse SH B. Both target slaughterhouse SH A and SH B utilize scalder and plucker for defeathering the chicken. The scalders use fossil fuel to generate hot water (40 and 58 °C of SH A and SH B, respectively), which is also source of GHG emission in slaughterhouse. In this study, slaughterhouse SH A and SH B apply various fossil fuels, result in difference of GHG emission in their own final products (shown in Table 11). Slaughterhouse SH A apply both natural gas and diesel, while SH B apply LPG only. Higher GHG emission factor of natural gas (2.63 kgCO_2_e/m^3^) and diesel (3.34 kgCO_2_e/L) cause more annual emission in SH A (654.588 ton CO_2_e) than SH B (450.312 ton CO_2_e, use LPG, emission factor 2.22 kgCO_2_e/L). In addition, the operation setting of scalder and plucker between target slaughterhouses cause more GHG emission difference.

**Table 11:** GHG emissions from fossil fuel of target slaughterhouse SH A and SH B.

Slaughter House	Fuel	value	unit	Emission Factor (kgCO_2_e/unit)	GHG emission (tCO_2_e)	Total GHG emission (tCO_2_e)
SH A	LNG	191,733	m^3^	2.63	504.258	654.558
	Diesel	45,000	L	3.34	150.300	
SH B	LPG	202,843	L	2.22	450.312	450.312

Diesel is high-carbon intensive organic mixture which provide high calorific value (8,400 kcal/L). Low-emission fuels such as natural gas and LPG provide the lower thermal energy than diesel (calorific value 8,000 kcal/m^3^ and 6,635 kcal/L). But the low-emission fuels generate less GHG emission and air pollutant (including nitro oxides and sulfur oxides). From point of total calorific value demanded of target slaughterhouses SH A and SH B, 1.91 × 10^9^ and 1.35 × 10^9^ kcal were provided via fossil fuel annually (shown in Table 12). The higher quantity of chicken slaughtered in SH B does not make more calorific value demand. Target slaughterhouse SH A consumed 191, 733 m^3^ of natural gas and 45, 000 L of diesel, brings 504.258 and 150. 300 ton CO_2_e GHG emission (shown in Table 11). Slaughterhouse SH B, on the contrary, only utilize LPG (202,843 L) and result in significantly less GHG emission (450.312 ton CO_2_e). We estimate the using quantity and GHF emissions of fuel alternation of slaughterhouse SH A under the some 1.91 × 10^9^ kcal calorific value demand (shown in Table 13). The result shows that using of low-emission as alternative fuels to replace diesel could bring less GHG emission to the slaughterhouse. LNG and natural gas bring 628.526 and 639.690 ton CO_2_e, both bring obviously less GHG emission than present condition (using diesel and natural, result in 654.588 ton CO_2_e).

**Table 12:** Unit calorific value of fossil fuel using in target slaughterhouses SH A and SH B.

Slaughter House	Fuel	value	unit	Calorific value coefficient (kcal/ unit)	calorific value (kcal)	Total calorific value (kcal)	Unit calorific value (kcal/kg)
SH A	LNG	191,733	m^3^	8,000	1.53 × 10^9^	1.91 × 10^9^	47.63
	Diesel	45,000	L	8,400	3.78 × 10^8^		
SH B	LPG	202,843	L	6,635	1.35 × 10^9^	1.35 × 10^9^	30.91

**Table 13:** GHG emissions of SH A's using alternative fossil fuels under the same calorific value conditions.

Alternative fuel	value	unit	Coefficient (kgCO_2_e/unit)	GHG emission (tCO_2_e)
Diesel	227,603	L	3.34	760.194
LNG	238,983	m^3^	2.63	628.526
LPG	288,148	L	2.22	639.690

Although LNG and natural gas have less environment impaction than diesel, they are still belonging to fossil fuel. Consider the purpose of fossil fuel is defeathering via heating, electrification or renewable fuel application are expected to reduce the GHG emission in the slaughterhouse in the future.

### GHG emissions from wastewater treatment between target slaughterhouse SH A and SH B

**Fig. 4:**
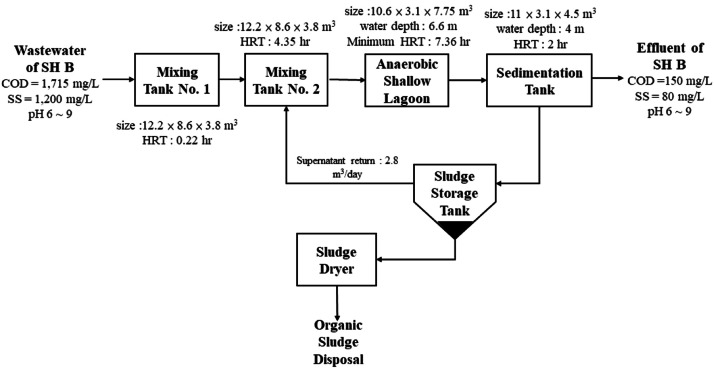
The wastewater treatment process of slaughterhouse SH B.

**Fig. 5:**
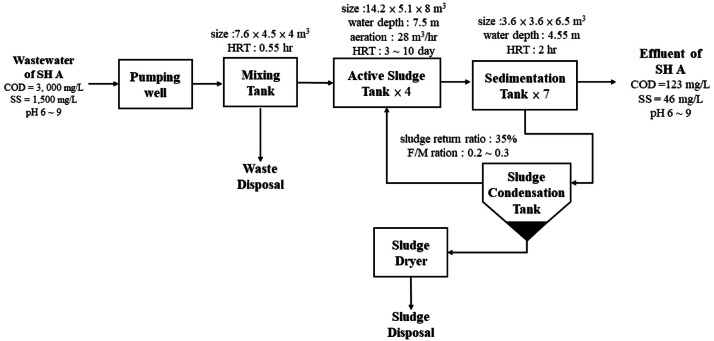
The wastewater treatment process of slaughterhouse SH A.

The wastewater stream from slaughterhouse presents a complex matrix characterized by high concentrations of organic matter, including amino acids, fats, peroxides, and hydrocarbon compounds derived from feces and leftovers. Additionally, it contains significant levels of nutrients such as nitrogen and phosphorus, along with suspended solids encompassing feathers, viscera, bones, and blood, all contributing to a high biochemical oxygen demand (BOD), chemical oxygen demand (COD) and suspended solid (SS). The presence of substances like blood, with its exceptionally high COD and SS, further exacerbates the pollution load and GHG emission during wastewater treatment. In this study, we select two typical electric slaughterhouses as targets for elucidating GHG emission of broiler product. the SH B was designed to operate rendering process that collect the feather from scalding for recycle application (the wastewater treating process is shown in Fig. 4). It brings fragment and debris of feather while washing the equipment and delivered into waste water facility in SH B result in more water usage but lower pollutant concentration (average COD = 1,715 mg/L, SS = 1,200 mg/L, shown in Fig. 4 and Table 14). On the other hand, target slaughterhouse SH A deliver all the organic matter into the wastewater treating facility (average COD = 3,000 mg/L, SS = 1,500 mg/L, shown in Fig. 5 and Table 14). The target slaughterhouse SH A applied aerobic decomposition method (four active sludge tanks, shown in Fig. 5) favored for lower energy requirements, less sludge production. Slaughterhouse SH B design the anaerobic digestion for lower residual organic matter in the in large volume of effluent (average COD = 150 mg/L, SS = 80 mg/L, shown in Fig. 4). Both the effluents of slaughterhouse SH A and SH B are complied with national effluent permit limit of slaughtering (COD = 150 mg/L, SS = 80 mg/L, Ministry of Environment, Taiwan).

**Table 14:** Wastewater condition of target slaughterhouse SH A and SH B.

Slaughter House	Item	value	unit
SH A	Annual wastewater treatment capacity	169,424	m^3^
	Average COD Concentration	3,000	mg/L
	Total COD	508,272	kg
SH B	Annual wastewater treatment capacity	380,179	m^3^
	Average COD Concentration	1,715	mg/L
	Total COD	652,006	kg

Power electricity and CH_4_ were calculated as the relevant GHG sources to both target slaughterhouses in this study. Wastewater treatment contribute 5.09 % of GHG emission comes from waste treatment (assumption of 2.63 % and 2.45 %, shown in Table 4, the last GHG emission sources) in slaughterhouse SH A but the second source in SH B (21.63 %, assumption of 4.28 % and 17.35 %, shown in Table 5). Although SH B has larger annual slaughtering amount (43,546,707 kg) and wastewater capacity (380,179 m^3^), but the COD concentration (1,715 mg/L in average) is relatively lower than SH A (annual slaughtering amount 40,136,095 kg, capacity 169,424 m^3^ and COD 3,000 mg/L in average). The slaughterhouse SH B has to digest 1.28 times more of total COD pollutant (652,006 kg) than SH A (508,272 kg) annually (shown in Table 14). This difference cause significantly more contribution of electricity consumption and GHG emission of slaughterhouse SH B than SH A (shown in Table 15).

**Table 15:** Comparison of treatment process and electricity consumption between target slaughterhouse SH A and SH B.

Item	SH A	SH B	Ratio (SH B/ SH A)
Feather chicken weight	40,136,095	43,546,707	1.08
Number of feather chicken	18,246,909	20,835,775	1.14
Total COD (kg)	508,272	652,006	1.28
Power consumption of wastewater treatment plant (kWh)	322,935	380,179	1.18

Methane is considered a major issue for GHG emissions in wastewater treatment. Wastewater contains a high concentration of organic matter. When this organic matter decomposes in the absence of oxygen (anaerobic conditions), specific microorganisms break it down and produce biogas, which is primarily composed of methane (typically 50-75 %). Simpler, less controlled anaerobic systems, common in some regions, allow almost all the produced methane to escape directly to the atmosphere. In the process of wastewater treatment at slaughterhouses, it is advisable to appropriately adjust the temperature, as an increase in temperature typically enhances the degradation efficiency of COD pollutant (Ahmad, et al., 2014). The methane correction factor (MCF, kg CH_4_/kg COD) is a crucial parameter in estimating methane emissions from wastewater treatment. It reflects the extent to which the methane-producing capacity of organic matter in wastewater is realized under different wastewater treating design and discharge conditions. The MCF is a fraction (range 0 ∼ 1) that indicates the degree to which a given wastewater treatment system or discharge pathway allows for anaerobic conditions, and thus, methane generation. A higher MCF indicates a greater potential for methane emissions. The Intergovernmental Panel on Climate Change (IPCC) provides guidelines and default MCF values for various scenarios. Traditionally, well-managed aerobic wastewater treatment facilities were often assumed to have zero methane emissions (MCF = 0). However, more recent research indicates that even well-managed aerobic systems can emit methane. In this study, slaughterhouse SH A consistently achieves high effluent quality, maintains effective pollutant removal (COD = 123 mg/L, SS = 46 mg/L, exceeding national effluent permit limits, shown in Fig. 5). Therefore, the MCF of slaughterhouse SH A was set at 0.05 for aerobic treatment process (the average of range 0 ∼ 0.1 for well-managed centralized aerobic treatment plant) according to IPCC 2006 Guidelines for National Greenhouse Gas Inventories and result in 177.260 ton CO_2_e methane emission annually. IPCC 2006 guideline default MCF as 0.2 to anaerobic shallow lagoon facility which depth less than 2 meters. In this study, for the slaughterhouse SH B, the MCF was set at 0.2 because of anaerobic unit has the 6.6 m water depth. Total 909.550 ton CO_2_e of methane emission was estimated for slaughterhouse SH B (shown in Table 16).

**Table 16:** Methane emissions and GHG emissions from wastewater treatment in target slaughterhouse SH A and SH B.

Item	SH A		SH B	
	value	unit	value	unit
Total COD	508,272	kg	652,006	kg
Methane correction factor (MCF)	0.05	kg CH_4_/kg COD	0.2	kg CH_4_/kg COD
In this study				
Methane production	6.353	ton	32.60	ton
GHG emission(Methane)	177.260	tCO_2_e	909.550	tCO_2_e
GHG emission (Power)	190.532	tCO_2_e	224.306	tCO_2_e
Total GHG emission	367.792	tCO_2_e	1,133.855	tCO_2_e
Unit GHG emission (kgCO_2_e/kg)	0.009	-	0.026	-
Unit GHG emission (kgCO_2_e/per chicken)	0.020	-	0.054	-
MCF = 0.3 (SH A assumed scenario) and 0.8 (SH B)				
Methane production	38.120	ton	130.40	ton
GHG emission(Methane)	1,063.559	tCO_2_e	3,638.20	tCO_2_e
GHG emission (Power)	190.532	tCO_2_e	224.306	tCO_2_e
Total GHG emission	1,254.091	tCO_2_e	3,862.506	tCO_2_e
Unit GHG emission (kgCO_2_e/kg)	0.031	-	0.089	-
Unit GHG emission (kgCO_2_e/per chicken)	0.069	-	0.185	-

CH_4_ is more than near 28 times as powerful potent as CO_2_ the powerful to contribute climate warming (GWP = 27.9, according to IPCC AR6 report, 2021). This characteristic of CH_4_ lead the design and operation of wastewater treatment facility are the key factors to reduce the GHG emission in the slaughterhouse (Kim, et al., 2023). In the case of slaughterhouse SH A, we set the MCF of SH A at 0.05 because of well aerobic facility management. If the facility is pollutant over-loaded and not-well managed, the MCF has to be raised to 0.3 (according to IPCC 2006 guideline), which may lead 6 times more methane emission in calculated significantly (assumed scenario, shown in Table 16).

For the slaughterhouse apply anaerobic wastewater treating facility, temperature is strongly related to methane emissions during anaerobic wastewater treatment, primarily because it directly impacts the activity and growth of the microorganisms responsible for methane production (called methanogens). Mesophilic range (25-45 °C, typically 30-40 °C optimal) is the most common operating range for anaerobic digesters. Within this temperature range, methanogens are highly active, leading to efficient organic matter degradation and high methane production rates. Mesophilic conditions are generally preferred for most anaerobic wastewater treatment applications. However, it may require larger reactor volumes or longer hydraulic retention time (HRT) compared to thermophilic systems to achieve the same level of organic pollutant digestion. The generation of CH_4_ is the major GHG emission source and must be carefully collected, covering the surface of anaerobic lagoon is recommended to decrease the direct emission of CH_4_ to atmosphere. In also, covering the anaerobic lagoon may decrease the air goes into digestion system and maintain the ideal treatment (McCabe, et al., 2014).

Slaughterhouse wastes can be converted into energy and hydrogen through proper technological conversion. Anaerobic digestion is very common and useful for the decrease of the higher demand for chemical and biochemical oxygen found in slaughterhouse effluent. During anaerobic treatment, the various fractions of slaughterhouse waste exhibit relatively high gas productivity due to their composition and high organic content. Among them, methane is an efficient and clean gaseous fuel for power generation. Originated from animal farm production, methane is renewable biogas source. The gas turbine driven by biogas may be implemented for electricity generation, which fulfill the demand of the circular economy and environmental protection (Chang, et al., 2019; Chowdhury, et al., 2022).

The reuse of by-products or waste is also an important strategy, such as convert animal fat to bio-diesel will have a positive impact on the environmental protection and economy. It is estimated near 4.8 million L of chicken fat oil could generate around 4 million L of biodiesel with the proper conversions of catalytic cycle of laccase and the transesterification process (Chowdhury, Nabi, Arefin, Rashid, Islam, Gudimetla and Muyeen, 2022). The new technological conversion to achieve future energy production is expected to be developed and integrated during the design and building stage of the slaughterhouse.

## Conclusion

In this study, two electrical slaughterhouses are selected to elucidate the GHG footprint contribution during chicken slaughtering. The result shows that electricity consumption comes from equipment utilization is the essential source of GHG emission. In addition, wastewater treatment facility, including aerobic and anaerobic, must be operated carefully to prevent methane from emission to atmosphere. The new technology to reduce the GHG emission is encouraged to be integrated into the slaughterhouse during design and building phase for maximizing the efficiency.

## Disclosures

All the named authors of this submitted manuscript “Contribution of greenhouse gas footprint of broiler product during electrical slaughtering in Taiwan” have no conflict of interest, financial or otherwise. There are no other relationships or activities that could appear to have influenced the submitted work.
